# Population genetic analysis of Chadian Guinea worms reveals that human and non-human hosts share common parasite populations

**DOI:** 10.1371/journal.pntd.0006747

**Published:** 2018-10-04

**Authors:** Elizabeth A. Thiele, Mark L. Eberhard, James A. Cotton, Caroline Durrant, Jeffrey Berg, Kelsey Hamm, Ernesto Ruiz-Tiben

**Affiliations:** 1 Biology Department, Vassar College, Poughkeepsie, New York, United States of America; 2 Parasitic Diseases Branch, Centers for Disease Control and Prevention, Atlanta, Georgia, United States of America; 3 Parasite Genomics Group, Wellcome Sanger Institute, Hinxton, United Kingdom; 4 The Carter Center, Atlanta, GA, United States of America; James Cook University, AUSTRALIA

## Abstract

Following almost 10 years of no reported cases, Guinea worm disease (GWD or dracunculiasis) reemerged in Chad in 2010 with peculiar epidemiological patterns and unprecedented prevalence of infection among non-human hosts, particularly domestic dogs. Since 2014, animal infections with Guinea worms have also been observed in the other three countries with endemic transmission (Ethiopia, Mali, and South Sudan), causing concern and generating interest in the parasites’ true taxonomic identity and population genetics. We present the first extensive population genetic data for Guinea worm, investigating mitochondrial and microsatellite variation in adult female worms from both human and non-human hosts in the four endemic countries to elucidate the origins of Chad’s current outbreak and possible host-specific differences between parasites. Genetic diversity of Chadian Guinea worms was considerably higher than that of the other three countries, even after controlling for sample size through rarefaction, and demographic analyses are consistent with a large, stable parasite population. Genealogical analyses eliminate the other three countries as possible sources of parasite reintroduction into Chad, and sequence divergence and distribution of genetic variation provide no evidence that parasites in human and non-human hosts are separate species or maintain isolated transmission cycles. Both among and within countries, geographic origin appears to have more influence on parasite population structure than host species. Guinea worm infection in non-human hosts has been occasionally reported throughout the history of the disease, particularly when elimination programs appear to be reaching their end goals. However, no previous reports have evaluated molecular support of the parasite species identity. Our data confirm that Guinea worms collected from non-human hosts in the remaining endemic countries of Africa are *Dracunculus medinensis* and that the same population of worms infects both humans and dogs in Chad. Our genetic data and the epidemiological evidence suggest that transmission in the Chadian context is currently being maintained by canine hosts.

## Author summary

Since the mid-1980’s, when Guinea worm (*Dracunculus medinensis*) was formally targeted for eradication, the associated national and international efforts to control and eliminate the parasite have been remarkably successful. As of 2017, 16 of the 21 countries with endemic transmission have been certified free of the disease by World Health Organization, and one country (Sudan) is in the pre-certification stage. However, recent and unprecedented prevalence of apparent Guinea worm infection in Chadian dogs has caused concern. That this seemingly sudden emergence in non-human hosts also coincided with an apparent reemergence of infection among humans in Chad after almost 10 years without reported cases raised questions about the population history of Guinea worm in Chad and whether worms from human and non-human hosts were, in fact, the same species. To address these questions, we characterized the genetic variation in Guinea worms collected from various host species and locations in Chad and in the other three endemic countries. Genetic variation was measured in adult female worms using sequence variation of mitochondrial DNA genes and repeat number polymorphism at 23 nuclear microsatellite loci. We found that, regardless of host species, all worms sampled from the remaining endemic countries in Africa are *D*. *medinensis* and show no evidence of isolated transmission on the basis of host species.

## Introduction

The international campaign to eradicate Guinea worm (*Dracunculus medinensis*) has made remarkable progress, reducing the annual number of cases from an estimated 3.5 million in the mid-1980s to 30 cases in 2017 [1, 2]. Of the 21 countries that had endemic transmission at the eradication campaign’s inception, 16 have been certified free of disease by WHO and one (Sudan) is in the pre-certification stage, having halted indigenous transmission as of 2002. Current efforts are focused on interrupting transmission in the remaining endemic countries of Chad, Ethiopia, Mali, and South Sudan. Particular attention is also being given to the recent occurrence of apparent Guinea worm infection in non-human hosts. Domestic dogs have been the most commonly encountered non-human host by a significant margin, but domestic cats (in Chad) and olive baboons (in Ethiopia) have also been found with emerging adult worms [3]. The incidence of dog infection has been most acute in Chad, with more than 500 infections reported annually since 2016 [2].

Dog infections in the African context were first noted by Eberhard et al. [4] when they investigated the apparent re-emergence of Guinea worm disease (GWD) in Chad following an almost 10-year absence of reported cases. That GWD appeared to stage a comeback in Chad has been attributed to a lack of adequate nationwide surveillance, as evidenced by four separate WHO certification team assessments finding that surveillance did not meet WHO standard requirements for declaring Chad free of transmission. But the co-occurrence of dog and other non-human host infections in this Chadian outbreak, along with seemingly novel epidemiology among humans, raised significant concerns. Chief among those concerns were questions regarding the source of both the human and non-human infections (endemic or introduced?) and about the status of the relationship between worms from human and non-human hosts. For example, are the parasites the same species and/or is the same parasite population responsible for infections in both human and non-human hosts? Initial genetic observations by Eberhard et al. [4] found no genetic difference between adult females collected from dog and human hosts at the 18S rRNA gene, which has previously been shown to distinguish among *Dracunculus* congeners [5]. However, given the highly conserved nature of the 18S rRNA gene, there was concern that it was an insufficiently sensitive tool for discerning cryptic speciation (i.e., genetically and biologically distinct species that are morphologically indistinguishable). Likewise, even in the absence of cryptic speciation, there is considerable interest in determining whether Guinea worms in dogs and humans are maintaining isolated transmission cycles, particularly given recent evidence supporting the role of paratenic and/or transport hosts in the Chadian Guinea worm life cycle [6–8].

This work aimed to further clarify both the reemergence of Guinea worm in Chad and, in particular, the relationship between parasites emerging from human and non-human hosts. Using sequence variation in four mitochondrial genes (*cytB*, *cox3*, *nd3*, and *nd5*) and length polymorphism of 23 nuclear microsatellites, we investigated the relationship among Guinea worms from the four endemic countries and between host species within Chad.

## Methods

### Sampling

The primary focus of this work was to evaluate the distribution of genetic variation between human and non-human hosts in Chad, where the occurrence of Guinea worm infection in non-human hosts has been most numerically intense. However, to assess whether the Chadian Guinea worm population is truly anomalous, we also included *D*. *medinensis* samples from contemporary cases in the other three endemic African countries of Ethiopia, Mali, and South Sudan, including specimens obtained from dogs in South Sudan and Ethiopia and an olive baboon (*Papio anubis*) in Ethiopia. Active village-based surveillance for Guinea worm infection is ongoing in at-risk areas in all four endemic countries. This entails multiple weekly household-by-household searches for cases, immediate actions to contain transmission by isolating the patient from contact with surface water sources, collecting the emerging worm, and reporting patent or suspected/symptomatic dracunculiasis cases to the national eradication program. Emerging adult female worms were collected from both human and non-human hosts during the course of standard Guinea worm surveillance and containment from 2014‒2016 and stored in ethanol as described in Eberhard et al. [4].

### Ethics statement

The active surveillance described above and manual extraction of emerging adult worms are the standard containment and treatment procedures for Guinea worm infections, as agreed upon and sanctioned by the World Health Organization and country ministries of health. All extractions were performed by trained program or ministry staff. Moreover, all worms allegedly emerging from skin lesions on human hosts must be lab tested by the WHO Collaborating Center for Research, Training, and Eradication of Dracunculiasis at the Centers for Disease Control and Prevention in Atlanta, GA, for case confirmation. Human case samples were anonymized prior to inclusion in this study.

Human DNA was collected from North American volunteers by cheek swab to serve as a mammalian DNA negative control to verify specificity of the molecular markers used in this study. All volunteer donors provided informed verbal consent to DNA provision. No donor information was collected, and cheek swabs were combined into a single “human sample” prior to DNA extraction to further anonymize the material. At no point in this study was sequence data generated for this or any human DNA sample.

### Molecular methods

Whole genomic DNA was extracted from 5-15mm sections of adult female worm tissue via standard cell lysis, protein precipitation, and ethanol precipitation. Briefly, tissue was incubated in cell lysis buffer (100mM Tris-Cl, pH 8.5; 10mM EDTA; 100mM NaCl; 1% SDS; 0.4mg/mL proteinase K; and 2mM dithiothreitol) for 2–3 hours at 65°C with occasional agitation, followed by protein precipitation with 8M ammonium acetate added to a final concentration of 2.5M. DNA was then separated from the aqueous supernatant via standard ethanol precipitation with the assistance of GlycoBlue Coprecipitant (45ug/mL final concentration; Thermo Fisher Scientific), dried, and resuspended in 100uL TE buffer (10mM Tris-Cl, pH8.0; 0.1mM EDTA, pH8.0). Final DNA concentration was estimated with a NanoDrop 1000 spectrophotometer (Thermo Fisher Scientific). Sham extractions were performed with each round of worm specimen extraction and included in downstream applications to serve as an extraction negative control.

To investigate mitochondrial variation within and among the African Guinea worm specimens, sequences were generated for three loci, which cover four mitochondrial genes: 1863bp spanning the entirety of the *nd3* and *nd5* genes, 647bp within the *cytB* gene, and 594bp within the *cox3* gene. Loci were amplified individually in 25uL reactions comprising 50ng DNA, 1X Q5 HiFi MasterMix (Qiagen), and 0.5uM of each primer using a “touchdown” cycling protocol to account for possible primer target degeneracy across the various worm origins (S1 Table). Cleaned amplification products (ExoSap [Applied Biosystems, New York, NY]) were sequenced in both directions with BigDye Terminator v3.1 cycle sequencing chemistry (Applied Biosystems) and analyzed on a 3130*xl* Genetic Analyzer (Applied Biosystems) at the Cornell University Biotechnology Resource Center. Electropherograms were visually inspected and assembled with ChromasPro v1.7.4 (Technelysium, South Brisbane, Australia). Assembled contigs for each locus were aligned in MEGA v7.0 [9] and any polymorphic sites were reviewed in the original electropherogram and assembly to verify the nucleotide assignment. Prior to data analysis, all sequences for each locus were translated to the protein sequence (using the invertebrate mtDNA code in MEGA v7.0) to verify amplification of coding genes, trimmed to a length common across all individual worms, and then concatenated to form a single mitochondrial sequence for each individual (3015bp final). In addition, partial *cox1* sequences were generated as above for a subset of 38 specimens from across the African geographical and host species range to allow congeneric comparisons with North American *D*. *insignis* and *D*. *lutrae* sequences accessioned in GenBank [10].

To investigate more recent parasite population history and fine-scale genetic patterns, repeat variation at tri- and tetranucleotide microsatellite loci was evaluated. A putative set of loci with pure tandem repeats was generated by an MSDB [11] query of the draft *D*. *medinensis* genome (v2.0.4) generated by the Wellcome Sanger Institute and available from WormBase ParaSite (https://parasite.wormbase.org/index.html) [12–14]. Forty-eight loci were screened for reliability of amplification and repeat length polymorphism using a subset of *D*. *medinensis* specimens representing the present geographic and host species range of the parasite. Human epithelial (cheek) DNA was used as a representative mammalian DNA negative control during screening to ascertain and verify primer specificity at each locus. A set of 23 polymorphic loci with highly repeatable peak profiles over duplicated sample runs and minimal allelic dropout was retained for final processing and population genetic analysis. Following the method reported by Blacket et al. [15], each locus-specific forward primer was modified with a 5′ universal primer sequence tail matching one of four fluorescently tagged universal forward primers to facilitate economical multiplexing of loci (S2 Table). To encourage uniform polyadenylation of amplification products and minimize genotyping error, the 5′ end of all reverse primers was “PIG-tailed” following Brownstein et al. [16] (S2 Table). Loci were amplified in 10uL multiplex reactions comprising 50ng genomic DNA, 1X Type-It Multiplex Mastermix (Qiagen), 0.5uM each of either 3 or 4 forward primers, 0.5uM each of the appropriate fluorescent universal primer, and 1uM of each reverse primer. PCR products were then further “pseudo-plexed” to a total of 6‒8 loci per reaction (as permitted by product size range and fluorophore color) prior to fragment analysis on a 3130*xl* Genetic Analyzer (Applied Biosystems) at the Cornell University Biotechnology Resource Center. Alleles were manually scored in PeakScanner v2.0 (Applied Biosystems). A subset of worm specimens were genotyped multiple times to verify peak patterns.

### Data analysis

At the time of emergence, female *D*. *medinensis* are essentially tubes of larvae with relatively little maternal tissue and few areas reliably free of larval tissue. Therefore, with the exception of adult segments where no larvae were observed, extracted DNA is a pool of maternal and larval genomic DNA. For mitochondrial sequence data this should not pose a problem, given expected maternal inheritance of the mitochondrial genome. Repeatably clean sequencing data observed during this work would support that assumption. However, a mix of maternal and paternal information will be captured during amplification of codominant nuclear markers such as microsatellites. Therefore, with DNA extracted from a gravid female, and assuming monogamous mating, we can expect to see up to 4 alleles per locus, rather than the 2 alleles expected given the diploid nature of the organism. For the purposes of performing population genetic analyses that utilize estimation of Hardy-Weinberg equilibrium (HWE), a putative maternal genotype was deconvoluted (derived) for each extraction using the mixture ratio estimation method described by Gill et al. [17] (Suppl. File 1). Reliability of deconvoluted maternal genotypes was evaluated with repeated amplification, fragment analysis, and deconvolution of a subset of individuals as mentioned above. In all instances of repeated genotyping and genotype deconvolution, the operator was blind to the previous results. To ensure statistical analyses were not skewed by the deconvolution process, they were repeated (where possible) with “pseudo-dominant phenotypes” generated from raw “pooled” genotypes using the methods of Mengoni et al. [18] and Rodzen et al. [19] for evaluating genetic relationships among polyploid organisms. Briefly, the raw “pooled” genotype of an individual is converted to a vector of binary states similar to an AFLP phenotype. For each locus, a vector of all alleles observed in the population is generated and, for each individual, presence of each allele is coded as 1 and absence as 0. Thus, for a given locus *j* with *n*_*j*_ alleles observed in a population, each individual will have a 1 x *n*_*j*_ vector of dominant markers. The markers at each locus are then concatenated to give a ∑_*j*_ *n*_*j*_ marker multilocus genotype for each individual.

Mitochondrial and derived maternal microsatellite gene diversity (*H*, [20]) of parasite populations was estimated in Arlequin v3.5 [21]. To account for the influence of disparate sample sizes on the likelihood of sampling unique alleles, allelic richness and number of alleles private to parasite populations were estimated using the rarefaction approach as implemented in the program ADZE v1.0 [22]. These measures were estimated for both the derived maternal microsatellite genotypes as well as for mitochondrial haplotypes. For the mitochondrial analysis, unique haplotypes for each gene used in the study (*cytB*, *cox3*, *nd3*, and *nd5*) were coded as alleles and combined to generate a 4-locus mitochondrial genotype for each individual.

Non-random association of parasite genotypes on the basis of host species and geographical location was evaluated with several methods. For descriptive purposes, patterns of pairwise genetic divergence were calculated for mitochondrial sequence data using the uncorrected pairwise proportion of nucleotide differences (*p-*distance) in MEGA7 with 1000 bootstrap replicates [9]. Patterns of microsatellite divergence were visualized with principal coordinates analysis (PCoA) in GenAlEx v6.5 [23] and with spatial principal components analysis (sPCA) in *adegenet* 2.0 [24, 25]. We investigated genetic structuring of parasite microsatellite genotypes among countries and within Chad using the Bayesian clustering analyses implemented in MavericK v1.0 [26] and BAPS v6.0 [27]. The clustering model used in MavericK is identical to that of STRUCTURE [28], but MavericK includes an implementation of thermodynamic integration (TI) [29–32] to estimate the marginal likelihood of alternative models of population structure for inference of the most likely number of subpopulations (*K*). To be clear, regardless of the method implemented, inference of the most-likely *K* was intended to evaluate degree of population structuring, not as a definitive estimate of subpopulation numbers. MavericK analyses were run for all available admixture models (admixture with fixed alpha = 1, admixture with variable alpha, and no admixture) to evaluate the posterior probability of each evolutionary model over *K* = 1‒20. For each run, the Markov chain Monte Carlo (MCMC) sampling was replicated 10 times with 1,000 burn-in iterations and 10,000 sampling iterations, and the TI estimator was run with 50 rungs, 500 burn-in iterations, and 1000 sampling iterations. Convergence and stationarity of the MCMC were assessed across all values of *K* with a trace plot of marginal log-likelihood versus sampling iteration. Model evidence was transformed to a linear scale and normalized to sum to 1 over all *K* in order to evaluate the posterior distribution of the *K* estimates in MavericK. Clustering analysis incorporating spatial information of samples (geographic location where an infected host was detected) was also performed using the spatial clustering of individuals model in BAPS v6.0 [33, 34] with 10 replicates of *k* = 2‒60. Finally, various groupings of parasites, including grouped by host species and a nested design of region (north vs. south of Manda National Park) and host species, were tested with analysis of molecular variance (AMOVA) in Arlequin using mitochondrial sequences, derived maternal microsatellite genotypes, and pseudo-dominant microsatellite phenotypes. In all AMOVAs, significance was tested with 5000 permutations of haplotypes, individuals, and populations among individuals, populations, and groups of populations [35]. In addition, the degree of population subdivision (on the basis of both host species and geography) was evaluated within Chad using pairwise measures of population differentiation (*F*_ST_) calculated in Arlequin. Significance was tested with 10,000 permutations of individuals or haplotypes among population groupings. For both AMOVA and tests of differentiation, statistical significance was set at *p* < 0.05.

Genealogical relationships between unique mitochondrial haplotypes were estimated with Bayesian inference as implemented in MrBayes v3.2.6 [36]. Prior to Bayesian MCMC analysis, the best partitioning scheme and models of evolution were selected in PartitionFinder v2.1.1, with the three codon positions of each of the four genes comprising the 12 data blocks [37, 38]. Using AICc (corrected Akaike Information Criterion) scores, the best partitioned model scheme was determined to be a combination of the HKY, HKY+I, and HKY+G models across codon positions (HKY: *cytB* position 1 and 3, all genes position 2, and *cox3* position 3; HKY+I: *nd3*, *cox3*, and *nd5* position 1; HKY+G: *nd3* and *nd5* position 3). Mitochondrial haplotypes were partitioned accordingly in MrBayes and all positions were unlinked to allow separate estimation of parameters and mutation rates. Gene trees were inferred with two independent, parallel MCMC analyses of four chains each. Runs of 1 million generations, with sampling every 500 generations and a relative burn-in of 25%, appeared sufficient to achieve convergence (average standard deviation of split frequencies < 0.01). Trees were visualized in FigTree v1.4.3 (Rambaut 2014; http://tree.bio.ed.ac.uk/software/figtree/) and converted to scalable vector graphics (SVG) format for final editing and annotation in Inkscape v0.92 (freely available at https://inkscape.org). Relationships among African and North American dracunculid species with *cox1* sequences were estimated in the same manner, using *Enterobius vermicularis* (GenBank EU281143) as an outgroup and mutation models F81, GTR, and HKY across codon positions 1, 2, and 3, respectively.

Given the apparently unique population history of Chadian *D*. *medinensis*, we performed an initial analysis of the Guinea worm demographic history using several methods. Specifically, we were interested in determining if any signature of population bottleneck or expansion (reflecting the case reporting history in Chad) could be detected in the molecular data. Deviation from neutrality and population decline/expansion were tested with Tajima’s *D* [39] and Fu’s *F*_*S*_ [40] for all country samples. Significance tests were based on 5000 simulations using the number of observed pairwise differences between mitochondrial haplotypes in Arlequin (significance *p* < 0.05). To account for the pronounced mutation rate heterogeneity of nematode mitochondrial DNA (and subsequent violation of the infinite sites model of evolution) [41], population history was also inferred via mismatch distribution analysis in Arlequin using Harpending’s raggedness index as the test statistic [42]. Deviation of the observed raggedness index from the null expectation of recent demographic expansion (smooth unimodal distribution with low raggedness) was tested with 1000 bootstrap replicates. Lastly, demographic history of Chadian Guinea worms was inferred with Bayesian skyline plot (BSP) analysis in BEAST2 [43, 44]. Sequences were partitioned as described above and the analysis was run under the assumption of a strict molecular clock using the reported *C*. *elegans* mitochondrial mutation rate of 1.57x10^-7^ mutations per generation [45] (i.e., per year for *D*. *medinensis* following the expected 1 year cycle of transmission), using the Jeffreys prior for population size. Following short run optimizations, four final chains were run for 20 million iterations each, with sampling every 2000^th^ iteration. Convergence of the MCMC and independence of samples (effective sample size [ESS] > 200) were verified by review of run logs in Tracer v1.6 (Rambaut et al. 2013, http://tree.bio.ed.ac.uk/software/tracer/).

## Results

### Genetic diversity

From 128 *D*. *medinensis* specimens collected from the four remaining endemic countries in Africa, complete concatenated mitochondrial haplotypes (3015bp) were generated for 118. Untrimmed, non-concatenated sequences are available in GenBank, accession numbers MH048098‒MH048448. Microsatellite genotypes comprising 18–23 loci were generated for 92 of these specimens. For both mitochondrial and microsatellite methods, failed reactions exhibited no association with geographic or host species origin of the specimen. Repeated amplification and genotyping of a subset of individual worm extractions (n = 66 repeated at least once) indicated that microsatellite amplification profiles were highly repeatable (mean standard deviation of relative peak height = 0.01, range: 0‒0.19). Due to our focus on the Chad Guinea worm outbreak and the higher prevalence of detected cases in Chad relative to the other three countries, Chadian *D*. *medinensis* were over-represented within the overall sample (64% and 67% within the mitochondrial and microsatellite datasets, respectively) (Table 1). Outside of the primary Chadian dataset, other non-human parasite specimens in the final dataset include parasites from one dog in South Sudan and eight dogs and one olive baboon in Ethiopia.

**Table 1:** Mitochondrial and microsatellite diversity statistics for parasites within chad (subdivided by host species) and among all four endemic countries.

	Within Chad			Among Countries			
	Human	Dog	Cat	Chad	Ethiopia	Mali	South Sudan
***Mitochondrial*** ***haplotypes***							
n	20	48	7	75	12	14	16
N_h_	14	15	3	24	6	4	4
S	66	69	18	77	14	22	7
*H*	0.96 (± 0.03)	0.85 (± 0.04)	0.67 (± 0.16)	0.88 (± 0.03)	0.85 (± 0.07)	0.67 (± 0.08)	0.52 (± 0.13)
π	0.006 (± 0.003)	0.005 (± 0.002)	0.003 (± 0.002)	0.005 (± 0.002)	0.002 (± 0.001)	0.003 (± 0.002)	0.001 (± 0.001)
A_R_	4.3 (± 0.2)	3.7 (± 0.2)	2.5 (± 0.5)	5.2 (± 0.4)	3.3 (± 0.8)	3.1 (± 0.4)	2.4 (± 0.2)
N_P_	0	0	0	4.1 (± 0.4)	2.2 (± 1.1)	2.2 (± 0.4)	1.4 (± 0.6)
Tajima’s D (*p*)	-0.41 (0.37)	-0.38 (0.42)	0.79 (0.81)	-0.27 (0.47)	-0.10 (0.50)	1.78 (0.98)	1.29 (0.93)
Fu’s *F*_S_ (*p*)	-0.02 (0.51)	4.16 (0.91)	5.13 (0.98)	1.44 (0.71)	0.75 (0.63)	7.72 (0.99)	2.52 (0.92)
*r* (*p*)*	**0.02** (0.77)^†^	0.09 (0.001)	0.64 (0.01)	0.03 (0.01)	**0.08** (0.64)^†^	0.56 (< 0.001)	**0.39** (0.10)^†^
***Microsatellites***							
n	12	44	6	62	11	10	9
N_a_	9.8 (± 3.3)	13.3 (± 5.0)	5.5 (± 1.3)	15.2 (± 5.9)	8.0 (± 2.5)	6.7 (± 2.0)	6.2 (± 1.7)
A_R_	6.2 (± 0.3)	5.5 (± 0.3)	4.7 (± 0.3)	7.0 (± 0.4)	6.1 (± 0.5)	5.7 (± 0.3)	5.3 (± 0.4)
N_P_	2.6 (± 0.3)	1.6 (± 0.2)	1.3 (± 0.2)	2.5 (± 0.3)	1.7 (± 0.3)	1.8 (± 0.3)	1.5 (± 0.3)
*H*	0.84 (± 0.43)	0.77 (± 0.38)	0.79 (± 0.43)	0.80 (± 0.39)	0.74 (± 0.38)	0.71 (± 0.37)	0.71 (± 0.38)
*H*_O_	0.68 (± 0.21)	0.70 (± 0.19)	0.82 (± 0.19)	0.71 (± 0.18)	0.78 (± 0.21)	0.49 (± 0.22)	0.60 (± 0.26)

Numbers in parentheses are standard deviations except where indicated. Bold text indicates statistical significance.

n, total number of parasites analyzed per group; N_h_, number of distinct haplotypes within each host group; N_a_, mean number of alleles per locus; S, number of segregating (polymorphic) sites; π, nucleotide diversity; A_R_, mean allelic richness per locus, standardized to the lowest n for a given genetic marker and population comparison (for mitochondrial data, unique haplotypes for each of the four genes used in the study were coded as alleles and combined to generate a 4-locus mitochondrial genotype for each individual); N_P_, mean number of alleles per locus that are private to each population (by host species within Chad or by country); *H*, Nei’s gene diversity (equivalent to the expected heterozygosity for diploid microsatellite data and the probability that two randomly chosen haplotypes are different for mitochondrial haplotype data); *H*_O_, observed heterozygosity

* and †: *r*, Harpending’s raggedness index of the observed mismatch distribution. Observed distributions that do not differ significantly from the expected distribution (*p* > 0.05) suggest recent population expansion.

**Fig 1:**
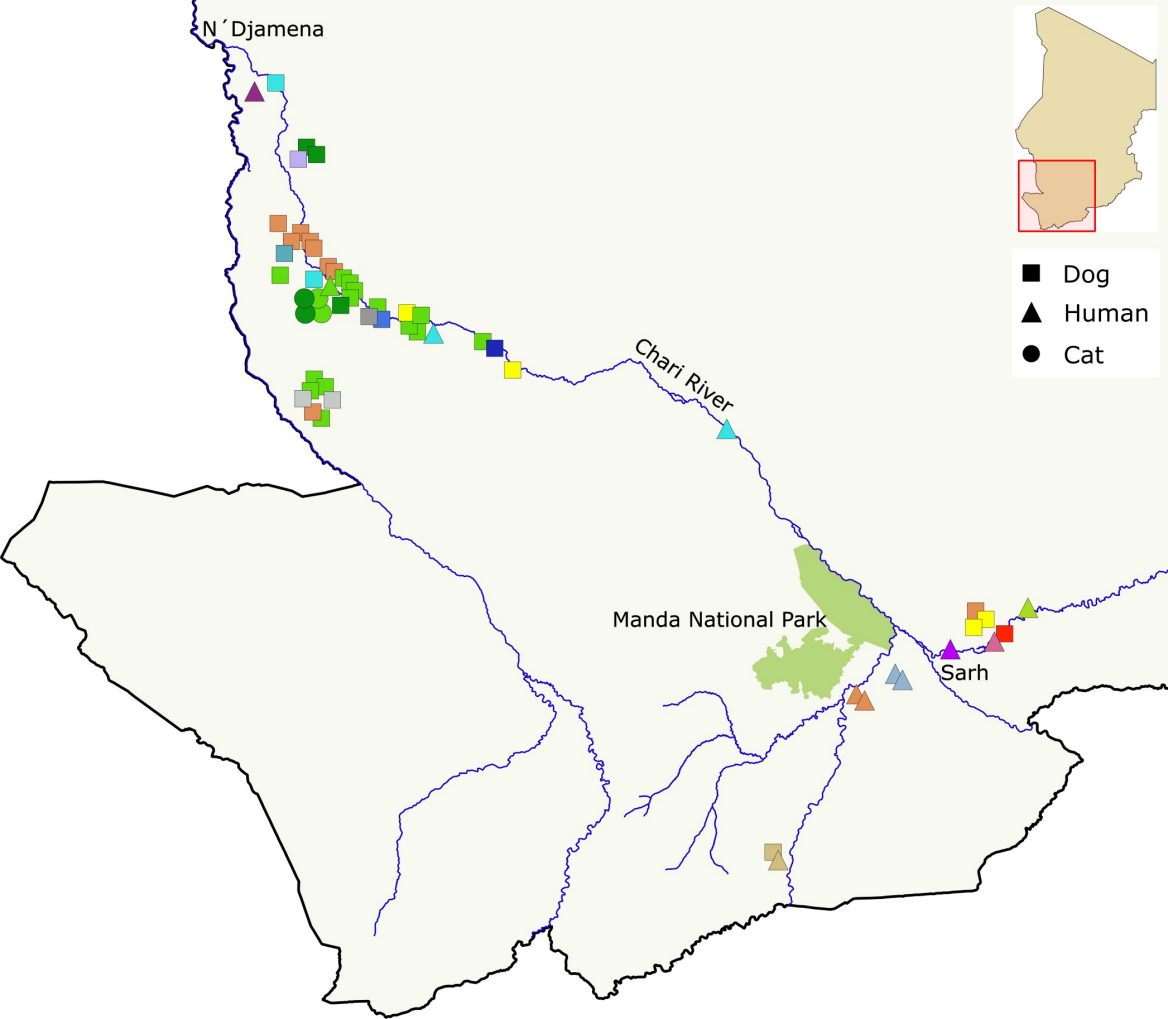
Distribution of mitochondrial haplotypes across Chad and among host species. Distribution of mitochondrial haplotypes across the geographic range and host species sampled in Chad. Host species are indicated by point shape and mitochondrial haplotype by color. The map was generated with QGIS v2.18.13 [46]. River paths and national park boundaries were extracted from Landsat 8 imagery provided courtesy of the U.S. Geological Survey (http://glovis.usgs.gov/).

Overall, the Chadian Guinea worm population was more diverse than the Malian, Ethiopian, or South Sudanese populations, with 24 unique mitochondrial haplotypes and high gene diversity (*H*_mtDNA_ = 0.88 ± 0.03). Microsatellite variation within the Chad population was also high, with an average of 15.2 (± 5.9) alleles per microsatellite locus (*H*_uSat_ = 0.8 ± 0.4) (Table 1). When correcting for sample size differences through rarefaction, the net difference in diversity between the Chadian population and other populations decreased, but Chadian *D*. *medinensis* remains the most diverse population in our sample (Table 1). Among Chadian humans, dogs, and cats, we find that mitochondrial and microsatellite diversity are highest in human and canine hosts with 9.8 (± 3.3) and 13.3 (±5.0) microsatellite alleles per locus (*H*_uSat_ = 0.84 and 0.77) and 14 and 15 unique mitochondrial haplotypes (*H*_mtDNA_ = 0.96 and 0.85), respectively (Table 1). Moreover, the number of microsatellite alleles private to a Chadian host species are generally comparable to levels observed among worms grouped by country of origin, while there were no mitochondrial haplotypes private to worms from any single host population within Chad (Fig 1).

### Distribution of genetic diversity

Mean overall pairwise divergence (*p-*distance) among concatenated mitochondrial haplotypes (*cytB-cox3-nd3-nd5*) from the 4 endemic countries was 0.5% (± 0.1%), with a mean intra-country divergence of 0.3% (± 0.1%; range: 0.1‒0.5%) and mean inter-country divergence of 0.5% (± 0.08%; range: 0.3‒0.5%) (Table 2). Among host species within Chad, the mean overall divergence was 0.5% (± 0.07%), with a mean intra-host divergence of 0.4% (± 0.09%; range: 0.3‒0.5%) that is not appreciably different from the mean inter-host divergence of 0.5% (± 0.04%; range: 0.4‒0.5%) (Table 2).

**Table 2:** Mean pairwise divergence (*p*-distance) of mitochondrial lineages within and between dracunculid parasites.

***D*. *medinensis–g*rouped by country of origin**				
	Chad	Ethiopia	Mali	South Sudan
Chad	0.005			
Ethiopia	0.005	0.002		
Mali	0.005	0.004	0.003	
South Sudan	0.005	0.003	0.005	0.001
***D*. *medinensis–*within Chad, grouped by host species**				
	Chad Human	Chad Dog	Chad Cat	
Chad Human	0.005			
Chad Dog	0.005	0.005		
Chad Cat	0.005	0.004	0.003	
**Divergence within and among *Dracunculus* spp. (*cox1* only)**				
	*D*. *medinensis*	*D*. *insignis*	*D*. *lutrae*	
*D*. *medinensis*	0.005			
*D*. *insignis*	0.09	0.001		
*D*. *lutrae*	0.11	0.09	0.004	

Uncorrected pairwise proportion of nucleotide differences (*p*-distance) estimated in MEGA7 (Kumar et al. 2016) with 1000 bootstrap replicates. Estimates within and among *D*. *medinensis* alone used 3015 bp of mitochondrial sequence (concatenated *cytB*, *cox3*, *nd3*, and *nd5* genes). Estimates within and among *Dracunculus* spp. use 496 bp of *cox1* sequence (*D*. *insignis* and *D*. *lutrae* sequences from [10]).

Diagonal, mean within-group *p-*distance; below diagonal, mean between-group *p-*distance

**Fig 2:**
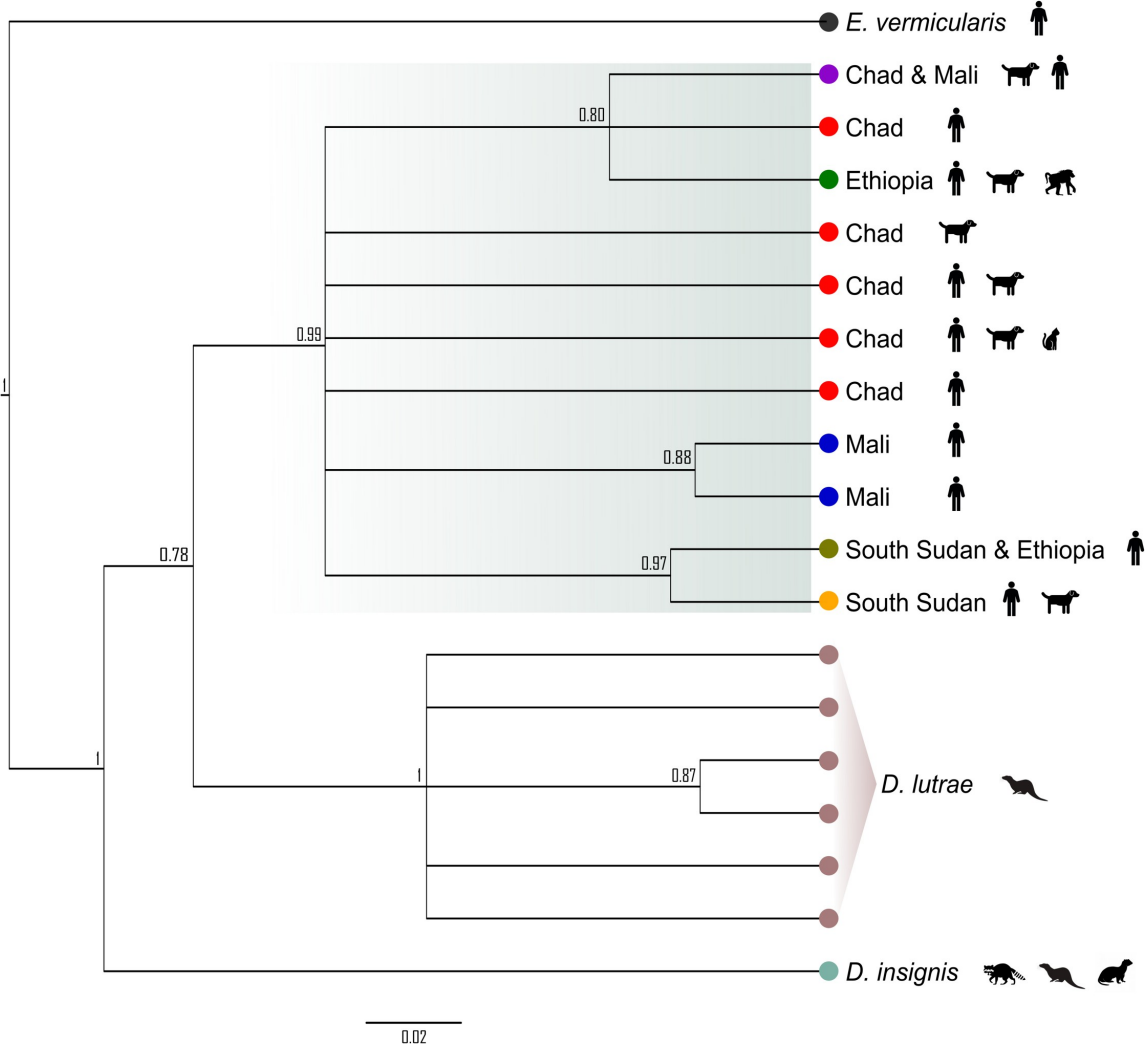
Phylogenetic relationship of African and North American *Dracunculus* spp. *cox1* haplotypes. Genealogical relationship among unique partial *cox1* haplotypes found in a subset of African *D*. *medinensis* and North American *D*. *insignis* and *D*. *lutrae* (North American species sequences from [10]). Country of origin (for African samples) and parasite species are defined beside each branch. Definitive host species from which all or some of the parasite haplotypes were recovered are indicated by icons to the right of the tree. Trees were inferred in MrBayes v3.2.6 [36] with two independent MCMC analyses of 1 million generations each. Posterior probabilities indicated at branch nodes.

**Fig 3:**
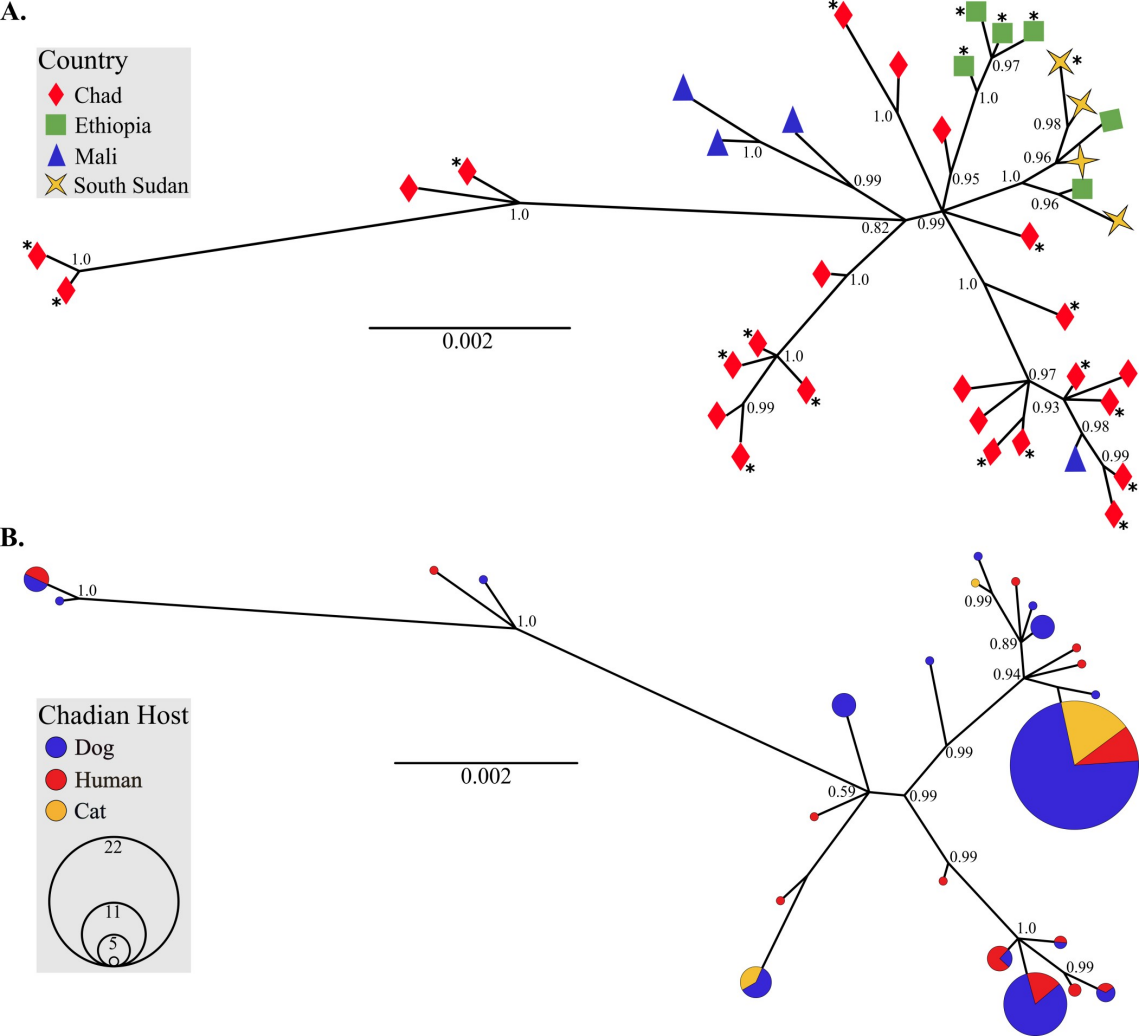
Phylogenies of mitochondrial lineages illustrate that African *D*. *medinensis* are a single species. (A) Genealogical relationship among unique *D*. *medinensis* haplotypes (concatenated *cytB*-*cox3*-*nd3-nd5*) from the four remaining endemic countries in Africa. Color and shape of tips indicate country of origin. Haplotypes where at least one parasite was collected from a non-human host are denoted with asterisks (*). (B) Relationship among unique *D*. *medinensis* haplotypes from Chad only. Circle size reflects prevalence of each haplotype in the Chadian population (smallest circles = 1) and color indicates the definitive host from which parasites were collected. Trees were inferred in MrBayes v3.2.6 [36] with two independent MCMC analyses of 1 million generations each. Posterior probabilities are indicated at branch nodes.

Using partial *cox1* sequences from North American *D*. *lutrae* and *D*. *insignis* and a subsample of the African *D*. *medinensis*, we found comparable levels of mean intra-specific sequence divergence in all three *Dracunculus* species (0.3% ± 0.2%). Divergence among species was significantly higher (average 10% ± 0.8%) and consistent with previous observations of interspecific divergence of congeneric nematode mitochondrial DNA [47] (Table 2). These intra- versus inter-host and intra- versus inter-specific divergence patterns are further borne out in genealogical evaluation of the mitochondrial haplotype relationships (Figs 2 and 3). The *cox1* gene tree (Fig 2) shows that all African parasites form a single, well-supported clade relative to the North American *Dracunculus* species. Both the partial *cox1* and concatenated mitochondrial gene trees illustrate that there is considerable overlap of host usage by Chadian parasites sharing the same mitochondrial haplotype and that there is no discernable pattern associated with definitive host usage (Fig 3).

**Fig 4:**
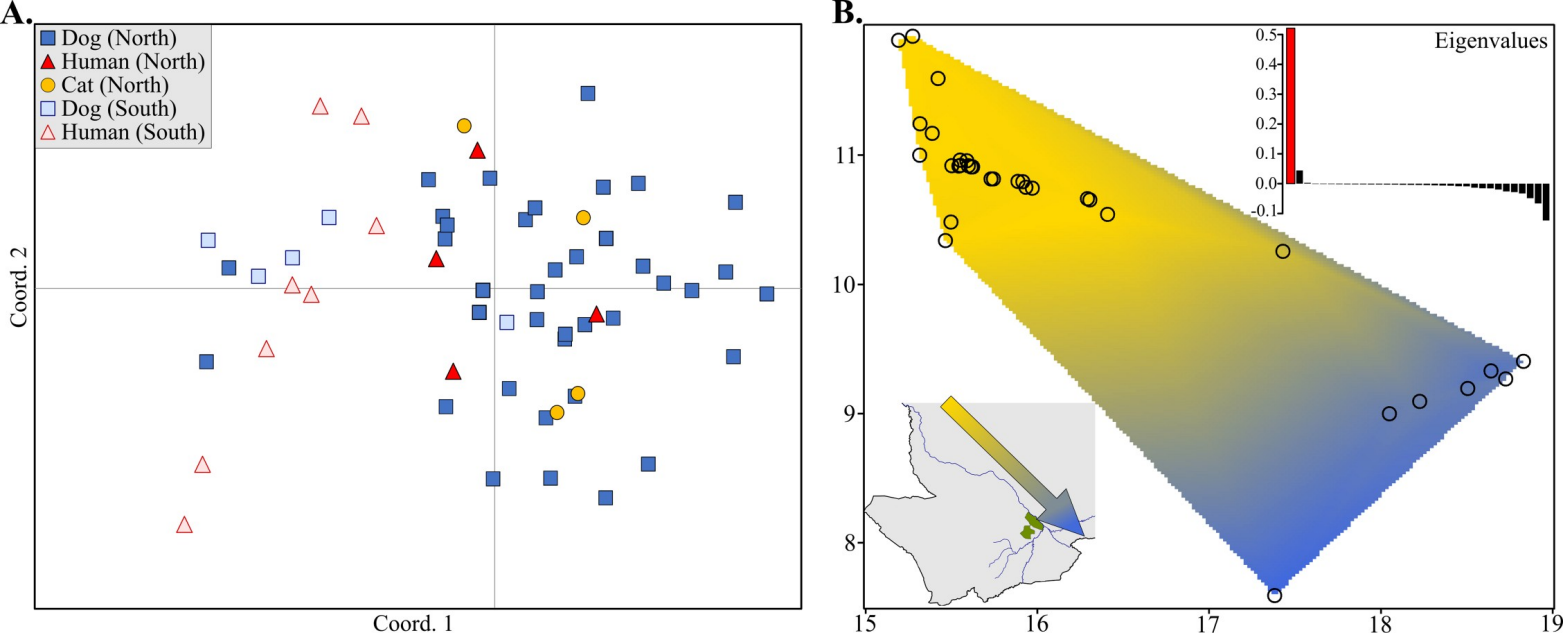
PCoA and sPCA suggest geographic differentiation of Chadian *D*. *medinensis* but no differentiation by host species. (A) PCoA of derived maternal genotypes for Chadian parasites. Point shape and color denote host species, and fill transparency indicates broad geographic origin of the parasite (northern or southern region). (B) Interpolation of lagged principal scores of the first component from the sPCA, plotted over the sampling area in Chad. Sampling locations of each parasite specimen are indicated by open circles, and genetic similarity is represented by color contours. The arrow in the lower left inset illustrates the clinal gradient in the context of the dominant geographic features of the sampling area. A barplot of the eigenvalues produced by the sPCA is shown in the upper right insert, illustrating the dominance of the first component within the sPCA.

Similarly, interrogation of microsatellite data with PCoA and sPCA found no evidence of genetic partitioning of parasites by host species in Chad (Fig 4). There was no clustering in PCoA that corresponded to differentiation on the basis of host species, though distribution of individuals along coordinate 1 suggested a possible geographic factor. The influence of geography on parasite differentiation was further supported by sPCA. The first (principal) component accounted for >50% of the variance, corresponding to genetic differentiation along a northwest to southeast gradient. Overlaid on a map of the sampling area in Chad, this suggested parasite clustering in regions broadly defined as being either northwest or southeast of Manda National Park (located just northwest of the city of Sarh along the Chari River).

**Fig 5:**
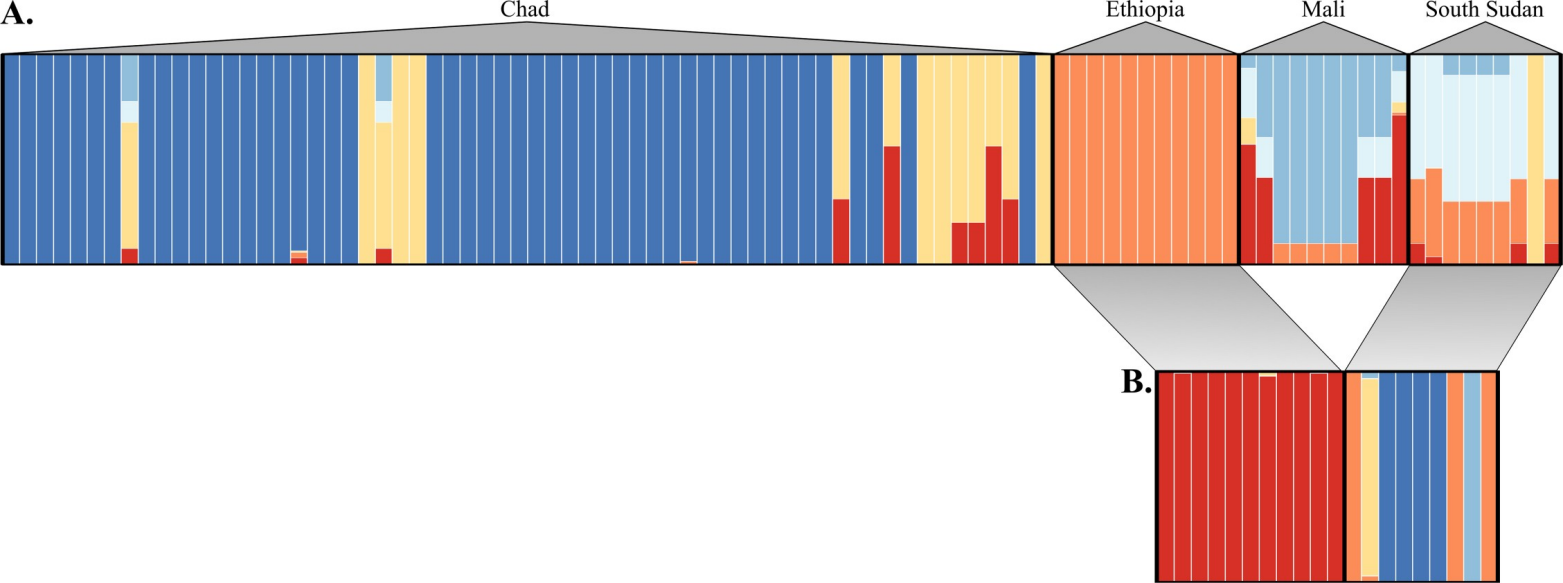
Inference of *D*. *medinensis* population subdivision among the four endemic countries. (A) Posterior assignment of individual *D*. *medinensis* worms collected from the four endemic countries into *K* = 6 clusters of shared ancestry. (B) Assignment of worms from only Ethiopia and South Sudan into *K* = 4 clusters of shared ancestry. In both analyses, each bar represents an individual worm and color indicates proportional assignment to one or more clusters. Bar colors are only informative within each assignment analysis, not between the two. Most likely *K* was inferred using the TI method in MavericK v1.0 [26].

**Fig 6:**
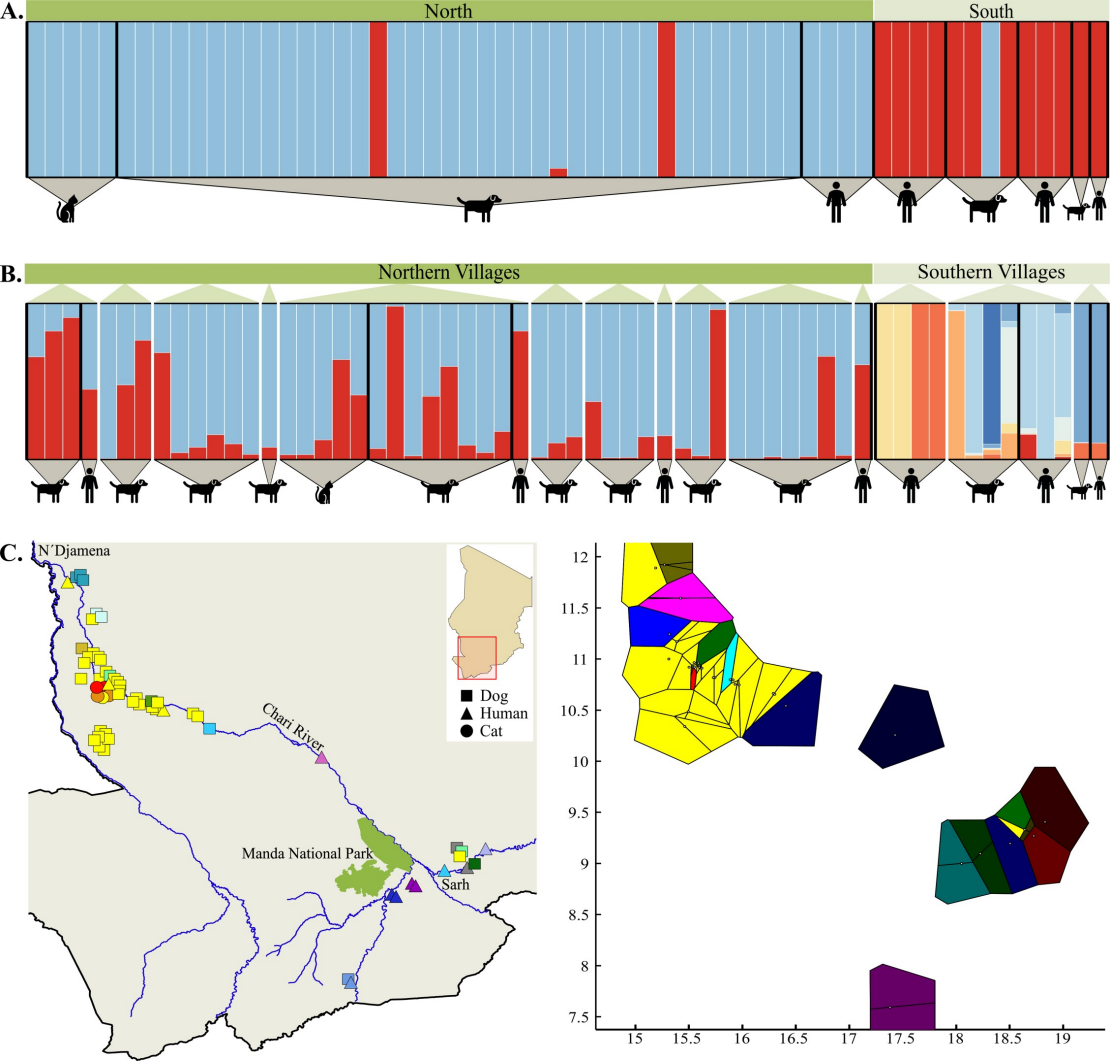
Inference of *D*. *medinensis* subdivision among host species and geographic regions within Chad. (A) Posterior assignment of all *D*. *medinensis* worms collected from within Chad into *K =* 2 clusters of shared ancestry, as inferred using the TI method in MavericK v1.0 to determine most likely *K* [26]. Each bar represents an individual worm and color indicates proportional assignment to one or more clusters. Individuals have been sorted by geographic region (i.e., north or south of Manda National Park) and definitive host species. (B) Assignment of worms when analysis is restricted by broad geographic region (i.e., north or south of Manda National Park) in MavericK v1.0. Northern worms were assigned into *K* = 2 clusters and worms from southern villages into *K* = 8 clusters. Within each geographic region, worms are sorted by village and definitive host species. Bar colors are only informative within each assignment analysis and should not be used for comparison among them. (C) Spatial clustering of individuals in BAPS v6.0 for most likely K = 16. Individual parasites plotted onto the map of the sampling area is depicted on the left, and Voronoi tessellation of clusters is illustrated on the right. Mapped point shape indicates host species and color indicates the cluster to which an individual was assigned. The map was generated with QGIS v2.18.13 [46]. River paths and national park boundaries were extracted from Landsat 8 imagery provided courtesy of the U.S. Geological Survey (http://glovis.usgs.gov/). Note that the cluster coloring scheme is not uniform between the mapped points and tessellation.

Bayesian inference of the distribution of microsatellite allelic diversity among parasite populations also indicated little to no genetic structuring on the basis of host species. Among countries, the data best fit the no admixture model, with *K* = 6 having the highest posterior probability (0.88 [95% CI: 0.68‒0.97]). Parasites collected from Ethiopian and South Sudanese hosts appear to have overlapping assignments in the all-country analysis, but inference with Ethiopian and South Sudanese parasites alone shows clear clustering on the basis of geographical origin (Fig 5). When evaluating all parasites sampled in Chad, the data best fit the no admixture model and *K* = 2 had the highest posterior probability (0.76 [95% CI: 0.66‒0.84]), with minor and significantly lower support for *K =* 3 (0.24 [95% CI: 0.16‒0.34]). Visual inspection of the Q-matrix plot indicated that the posterior probabilities of individual assignments to clusters were not associated with the parasite’s definitive host species, regardless of the level of *K*. Corroborating the PCoA and sPCA results, assignment of parasites to clusters tended to correspond to geographical origin of the parasites (as either north or south of Manda National Park). Subsequent evaluation of structuring within the North and South geographic sub-groups again indicated no clear shared ancestry on the basis of definitive host in either region (Fig 6A and 6B). Analyses performed in STRUCTURE v2.3 with the pseudo-dominant microsatellite phenotypes [28, 48] resulted in qualitatively equivalent results. Finally, explicit inclusion of geographic data via spatial clustering of individual Chadian parasites with BAPS v6.0 corroborated the findings of PCoA, sPCA, and MavericK. Spatial clustering in BAPS suggested a most likely *K* = 16, with geographic origin of parasites, again, being a better predictor of cluster assignment than host species (Fig 6C).

AMOVA using both mitochondrial sequences and microsatellite genotypes (derived maternal and pseudo-dominant phenotypes) corroborated the genetic structuring inferred with Bayesian analysis (Table 3). Within Chad, when evaluated solely on the basis of host origin, variation among host species populations accounted for only 3‒4% of the molecular variance (derived microsatellite genotype and mitochondrial sequence *p* > 0.07, pseudo-dominant phenotype *p* < 0.001). When a nested scheme was implemented in response to evidence of a broad regional subdivision in Chad, the percentage of variance accounted for by among-host species groupings was reduced to 1‒4% (mitochondrial sequence *p* = 0.23, all microsatellite *p* ≤ 0.03). Regardless of the subdivision scheme tested, variation within individual parasites or among worms from different hosts within a host species accounted for a significant majority of the variance (80‒96%, Table 3). Pairwise *F*_ST_ measurements among hosts and regions further corroborate the observed patterns of population differentiation dominated by geographic origin (Tables 4 and 5). Mean pairwise *F*_ST_ among hosts within regions was 0.03 (± 0.03), 0.02 (± 0.004), and 0.05 (± 0.02) for mitochondrial haplotypes, derived maternal microsatellite genotypes, and pseudo-dominant microsatellite phenotypes, respectively. The only significant intra-region, inter-specific differentiation was that of northern dog versus northern cat hosts for both iterations of the microsatellite genotype (*F*_ST_ 0.02 and 0.06, *p* = 0.04 and < 0.001, respectively). Mean pairwise *F*_ST_ among regions were higher (0.26 ± 0.13; 0.06 ± 0.02; and 0.11 ± 0.03 for mitochondrial haplotypes, derived microsatellite genotypes, and pseudo-dominant microsatellite phenotypes, respectively) and almost all significant at *p* < 0.05 (Tables 4 and 5).

**Table 3:** Analysis of molecular variance (AMOVA) of Chadian *D*. *medinensis* among host species and broad geographic origin.

Grouping	N	Variance Components	% of Variation	*p* - value
***Mitochondrial haplotypes***				
Within Chad by host species	3	Among host species	3.7	0.09
		Among hosts within a species	96.3	N/A
Within Chad by region* & host species	6	Among regions	18.7	0.1
		Among host species within regions	1.0	0.23
		Among hosts within species and region	80.3	0.005
***Microsatellites – derived maternal genotypes***				
Within Chad by host species	3	Among host species	2.7	0.07
		Among hosts within a species	9.3	<0.001
		Within individual worms	88.0	<0.001
Within Chad by region* & host species	6	Among regions	4.1	0.1
		Among host species within regions	1.8	0.03
		Among hosts within a species & region	7.9	<0.001
		Within individual worms	86.3	<0.001
***Microsatellites – pseudo-dominant phenotypes***				
Within Chad by host species	3	Among host species	5.6	<0.001
		Among hosts within a species	94.5	N/A
Within Chad by region* & host species	6	Among regions	8.8	0.1
		Among host species within regions	3.9	0.01
		Among hosts within a species & region	87.3	<0.001

Statistical significance determined by 5000 permutations of haplotypes, individuals, and populations among individuals, populations, and groups of populations in Arlequin 3.5.

* Regions are defined as north and south of Manda National park.

N, number of groups being evaluated; N/A, statistic not estimated in this analysis

**Table 4:** Pairwise *F*_ST_ among mitochondrial haplotypes and pseudo-dominant microsatellite phenotypes in Chadian *D*. *medinensis*.

		North			South	
		Cat	Dog	Human	Dog	Human
North	Cat		**0.06**	0.08	**0.11**	0.16
	Dog	0.01		0.04	**0.11**	**0.14**
	Human	0.08	0.03		**0.06**	**0.09**
South	Dog	**0.38**	0.13	0.14		0.01
	Human	**0.47**	**0.19**	**0.24**	0.0	

Bold values indicate statistical significance at *p* < 0.05.

Below diagonal, pairwise *F*_ST_ of mitochondrial haplotypes; above diagonal, pairwise *F*_ST_ of microsatellites as pseudo-dominant phenotypes

**Table 5:** Pairwise *F*_ST_ Among derived maternal microsatellite genotypes in Chadian *D*. *medinensis*.

		North			South	
		Cat	Dog	Human	Dog	Human
North	Cat		0.08	0.14	0.27	0.41
	Dog	**0.02**		0.07	0.24	0.35
	Human	0.03	0.02		0.16	0.27
South	Dog	**0.05**	**0.04**	0.02		0.03
	Human	**0.09**	**0.08**	0.06	0.02	

*Bold values indicate statistical significance at p < 0*.*05*.

Below diagonal, pairwise *F*_ST_ of derived maternal microsatellite genotypes; above diagonal, pairwise standardized *Fʹ*_ST_ of derived maternal microsatellite genotypes (statistical significance is not assessed for this measure).

### Population history

**Fig 7:**
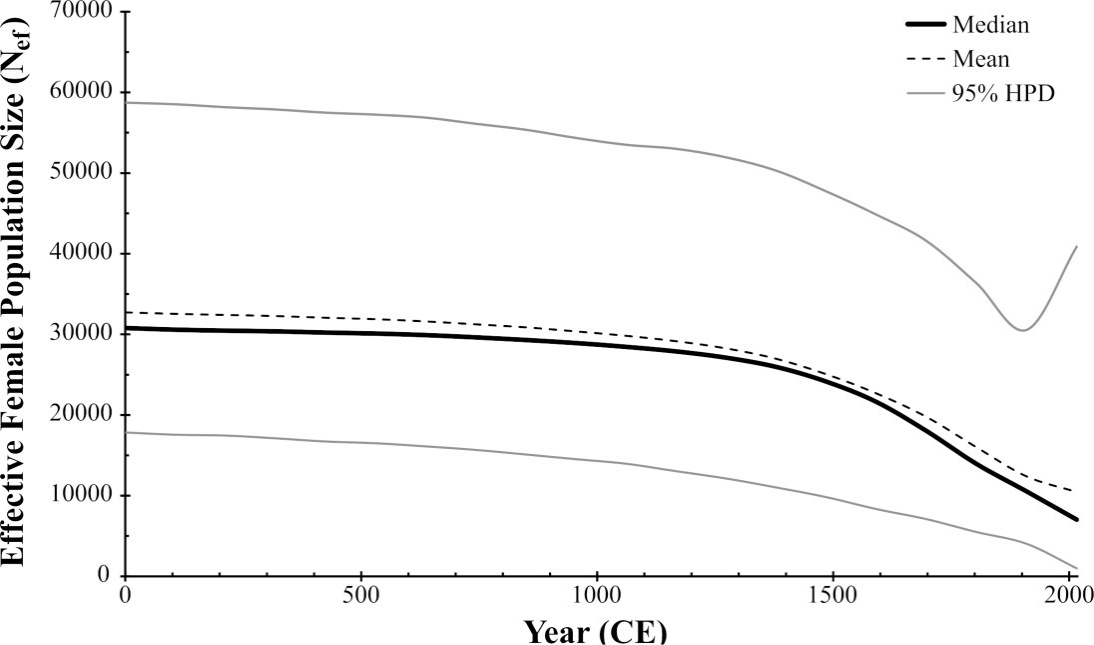
Bayesian skyline plot of the Chadian *D*. *medinensis* population over time. Derived from Chadian *D*. *medinensis* mitochondrial sequences (concatenated *cytB-cox3-nd3-nd5*, sampled from 2014‒2016). The x-axis is in years (0 to 2016 CE) and the y-axis is *N*_*eτ*_ (the product of the effective population size of female parasites and the generation time). Assuming a generation time of 1 year, this is equivalent to the effective population size of female parasites *N*_*ef*_. Analysis assumed a strict molecular clock and mutation rate of 1.57 x 10^−7^ mutations per site per generation.

As a whole, the Chadian Guinea worm population did not significantly deviate from neutrality by any measure (*D* = -0.16, *p* = 0.51; *F*_*S*_ = 1,83, *p* = 0.76; *R* = 0.03, *p* = 0.02) (Table 1). When subdivided by region, the subpopulation north of Manda National Park also indicated no deviation from neutrality (*D* = 0.02, *p =* 0.59; *F*_*S*_ = 4.55, *p =* 0.91; *R* = 0.06, *p* = 0.01). The southern subpopulation did not deviate from neutrality by either Tajima’s *D* or Fu’s *F*_*S*_ (*D* = -0.78, *p* = 0.23; *F*_*S*_ = 0.3, *p =* 0.57), but the mismatch distribution of southern mitochondrial DNA variation could not be differentiated from the null distribution model of population expansion (*R* = 0.02, *p* = 0.86). Demographic reconstruction of the Chadian Guinea worm history with BSP analysis in BEAST2 indicated a decline in the effective population size of female worms over the past ~600 years, but there is no signature of either a drastic bottleneck and/or expansion (Fig 7).

## Discussion

Both mitochondrial and nuclear data support the conclusion that Guinea worms collected from non-human hosts in this study are the same species as those collected from humans. Moreover, the current dataset does not suggest that Chadian parasite transmission is subdivided by host species. First, the maximum mitochondrial sequence divergence (*p*-distance) among parasites collected from different definitive hosts in Chad (0.5%) was effectively indistinguishable from the *p*-distances observed among parasites collected from within the same host species, as well as among all parasites from the four countries sampled in this study. This level of mitochondrial sequence divergence is on the low end of the range observed within populations of conspecific nematode parasites [47, 49] and well below that observed among congeners. Even among morphologically identical cryptic nematode species, mitochondrial sequence divergence has ranged from 8‒11% [50, 51]. Second, inferred genealogical relationships among mitochondrial haplotypes collected from all four countries and within Chad alone clearly indicate that, with a few exceptions, parasites tend to cluster by geographic origin but do not form private clusters on the basis of host species. Likewise, genealogical inference among the African and North American *Dracunculus* species show the African specimens, regardless of country or host species origin, forming a well-supported monophyletic clade. Finally, the distribution of variation among 23 nuclear microsatellite loci clearly corroborates that of the mitochondrial observations. Bayesian inference of population structure, PCoA, sPCA, and AMOVA all suggest that geographic origin of the parasite (e.g., whether a host resides to the north or south of Manda National Park in Chad) has a greater influence on parasite subdivision than definitive host species. And despite the influence of geography, the majority of genetic variation in Chadian parasites is found within conspecific hosts from the same region. The spatial clustering analysis produced by BAPS does show a higher degree of population subdivision than that of MavericK (or STRUCTURE with pseudo-dominant phenotypes). However, the increase in structuring still does not result in a pattern of partitioning by host species, likely reflects the uncertainty associated with low values of *F*_ST_ and differences in the algorithms by which *K* is estimated, and is consistent with previous reports of a tendency for BAPS to overestimate *K* [52].

Overall, the Chad Guinea worm population appears to have maintained a great deal of genetic diversity relative to the three other countries with continued endemic transmission. This observation lends credence to the conclusion that the almost decade-long period of zero case reporting in Chad prior to 2010 was due to insufficient surveillance rather than an absence of infection. It also suggests that the Chadian parasite population was not significantly constricted during that time. Mismatch distribution analysis in Chad’s southern parasite group did correspond to the distribution expected under population expansion, but as the southern population was less represented within this dataset, it remains to be seen if this pattern persists with the addition of data. Genealogical analyses (both in MrBayes and the coalescent process in the Bayesian Skyline analysis) infer a deep coalescence of the Chadian Guinea worm population. This can suggest a historically large, stable population (Ballard and Whitlock 2004) or the influx of individuals from differentiated populations. The latter scenario is not currently supported by our data, as genealogies constructed with parasites from all 4 endemic countries do not reflect recent immigration of parasites into Chad from either Mali, Ethiopia, or South Sudan to the extent that it would generate the observed coalescent depth. We cannot exclude the possibility that unsampled (and unobserved) neighboring Guinea worm populations have contributed to the mitochondrial variation observed in Chad, but the distribution of current microsatellite variation would suggest that any such immigration was more historical than recent. Moreover, genetic patterns observed here corroborate epidemiological patterns and case-study findings indicating that the apparent re-emergence of dracunculiasis in Chad was not due to a single point-source outbreak [53]. Ultimately, the demographic analysis and any estimated population sizes should be treated with caution at this point. First, the BSP methods employed assume that the mitochondrial mutation rate of *D*. *medinensis* is not significantly different from that of *C*. *elegans*. Given the short timespan of our sampling, we have little power to calibrate the rate (or pattern of variation in rate) for *D*. *medinensis*. Therefore, both the effective female population size and timeline estimates provided by the analysis should be treated as relative numbers, rather than absolute. Second, the sampling scheme, while attempting to be inclusive of both the parasite’s current geographical and host species range within Chad, was directed more at the question of host-specificity. This broad sampling may have resulted in an underrepresentation of some haplotypes, inflating estimates of *N*_*ef*_ [54]. Likewise, the coalescent analysis involved in BSP assumes that samples have been drawn from a single panmictic population [43]. Scattered sampling across a species range when populations are subdivided but maintain some gene flow (as would be suggested by analyses of genetic differentiation here) has been shown to produce false bottleneck signals in simulations. Thus, the magnitude of apparent decline in the more recent populations should be treated with caution [55–57].

That dogs could be serving as “maintenance hosts” [58] within the Chadian context appears highly likely. In addition to lack of genetic isolation of parasites among host species, the sheer prevalence of infection in dogs relative to humans [3, 59] would suggest that the dog population is capable of sustaining transmission in the absence of human infections. Additionally, despite the rarity of reported human cases, genetic patterns suggest that individual dogs and/or dogs from the same village are encountering larvae from multiple uncontained infections within their environment during a single transmission season. In the samples examined here we found that single hosts with multiple emerging worms almost always harbored multiple maternal lineages of parasite, suggesting the potential for high mitochondrial haplotype diversity at the local scale. We cannot entirely discount the possible role of unreported and uncontained cases within the human population in this situation. However, the dramatically increased surveillance efforts since 2011 [3, 4] and considerable monetary reward for reports leading to a contained case (approximately 100USD) would suggest that unreported human cases are likely rare. Therefore, undetected human cases, alone, would have insufficient force of infection to maintain the size and local genetic diversity of the parasite population within dogs. Moreover, the very sporadic nature of cases among humans (highly dispersed along the endemic area of the Chari River, no expansion of cases among village cohabitants in the years immediately following a human case, and no association with a common water source) is unique in the history of Guinea worm epidemiology. This can be interpreted as evidence of successful containment of reported cases in humans and of the theory that human cases in Chad now represent incidental spillover from the dog population. This interpretation is also supported by the genetic patterns observed here, but, given the broader species-level focus of the current study, sampling was not sufficient at local scales to rigorously address the more granular patterns of parasite distribution. Scaled up sampling efforts and genetic analysis of *D*. *medinensis* is currently under way to formally address questions of local parasite population dynamics within Chadian dogs.

Finally, “why here and why now?” is the natural next question and one that we may not be able to definitively answer. However, we can be reasonably certain that this does not represent a novel host switch. Infections in domestic dogs and cats have previously been reported, both as experimental hosts [60–63] and as natural incidental hosts [64–72]. Thus, while the observation of parasites emerging from non-human hosts may appear sudden in the African context, it is not novel within the history of the parasite. Dogs appear to be particularly receptive to *D*. *medinensis*. Muller commented that dogs seemed to be the most “popular” laboratory host for Guinea worms and reported that the primary limiting factor in laboratory maintenance of the life cycle is not lack of a suitable definitive host, but maintenance of viable copepod colonies [60]. And while the data presented here do not include direct evidence of human to non-human transmission (or vice versa), we point out that all previous assessments of dogs’ suitability as laboratory hosts utilized larvae collected from Guinea worms emerging from human hosts [60, 62, 63]. In addition, we now have specimens collected from non-human hosts in every remaining endemic country–this study includes worms collected from dogs and a baboon in Ethiopia and a single dog in South Sudan. We did not explicitly test the distribution of parasite genetic diversity among human and non-human hosts in these two countries because of limited sample size and statistical power. However, parasites collected from the South Sudanese and Ethiopian non-human hosts either share haplotypes with parasites collected from human hosts within the same country or, like the Chadian worms, are not sufficiently divergent in either mitochondrial or microsatellite variation to suggest the presence of a cryptic species. Thus, the primary difference between Chad and the other three endemic countries currently appears to be the respective roles of human and non-human hosts in parasite transmission. The underlying basis for these differences is a topic of concern with immediate and important practical implications but beyond the scope of this paper. The roles of dog behavior and resource usage are of special interest and being actively explored. Moreover, initial field and laboratory studies suggest a potentially novel ecological and epidemiological context in which amphibious and aquatic vertebrates could be facilitating Guinea worm transmission as paratenic or transport hosts [6–8]. Understanding how factors associated with aquatic ecology may be driving or supporting Guinea worm transmission in Chad is of particular importance, given that the Chari River and its floodplain are crucial sources of economic and dietary subsistence in the affected region of the country.

### Conclusion

Prior to the outbreak in Chad, reports of Guinea worm infection in non-human hosts were rare and based solely on the morphological and life history features unique to the parasite. This work shows that the hanging worms collected from non-human hosts in the remaining African foci of transmission are the same species of parasite as that infecting humans, *Dracunculus medinensis*. Moreover, we find no evidence of parasite subdivision that would suggest host-specific transmission patterns within Chad. The fact that no species-specific patterns of transmission have been observed here does not rule out the potential for isolation of transmission, either by targeted intervention or natural ecological isolation in resource usage–particularly for less household-integrated vertebrate hosts like domestic cats or truly sylvatic hosts like baboons. We are hopeful that ongoing studies to further elucidate transmission dynamics, such as more local population genetic studies, monitoring movement and resource usage patterns in non-human hosts, and modeling underlying eco-epidemiological patterns, will prove useful in isolating and ultimately eliminating transmission.

## Supporting information

S1 Table Primer sequences and thermocycling protocol for *D. medinensis cytB, cox3, nd3, and nd5* Genes. Melting temperature (T_m_) was calculated using the Q5 High-Fidelity DNA polymerase specifications in the NEB Tm Calculator (New England Biolabs). Melting temperatures are not provided for internal primers as they are only utilized in BigDye sequencing reactions. Note that *nd3* and *nd5* were amplified as a single unit.
(DOCX)

S2 Table Microsatellite primers and fluorescently tagged universal primer tails. All primers specific to *D*. *medinensis* (those whose names are prefaced with “Gw”) include a 5ʹ tail that is complementary to one of 4 fluorescently tagged universal primers, following Blacket et al. (2012). Microsatellite repeat motifs are based on the draft *D*. *medinensis* genome v2.0.4. Bolded text at the 5ʹ end of the reverse primers indicates additional PIG-tailing nucleotides appended following Brownstein et al. (1996).
(DOCX)

S1 File Derivation of maternal genotypes after amplification of pooled DNA extractions. (DOCX)
