# Supplementary material for: Population genetic analysis of Chadian Guinea worms reveals that human and non-human hosts share common parasite populations
Source: PLoS Negl Trop Dis. 2018 Oct 4;12(10):e0006747. doi: 10.1371/journal.pntd.0006747 (PMC6191157; doi:10.1371/journal.pntd.0006747)
Supplement: S1 Table — Melting temperature (Tm) was calculated using the Q5 High-Fidelity DNA polymerase specifications in the NEB Tm Calculator (New England Biolabs). Melting temperatures are not provided for internal primers as they are only utilized in BigDye sequencing reactions. Note that nd3 and nd5 were amplified as a single unit. (DOCX) [file pntd.0006747.s001.docx]

**Supplemental Table 1**

| **Suppl. Table 1.** **Primer Sequences and Thermocycling Protocol for *D. medinensis cytB, cox3, nd3, and nd5* Genes.** Melting temperature (T_m_) was calculated using the Q5 High-Fidelity DNA polymerase specifications in the NEB Tm Calculator (New England Biolabs). Melting temperatures are not provided for internal primers as they are only utilized in BigDye sequencing reactions. Note that *nd3* and *nd5* were amplified as a single unit. | | | | | | |
| --- | --- | --- | --- | --- | --- | --- |
| Primer | T_m_ (°C) | | Sequence (5ʹ→3ʹ) | | |  |
| cytB_F_EXT | 57.8 | | TGTTTAAGTATTTGAATTCTATGGTGGT | | |  |
| cytB_R_EXT | 58.2 | | GGTCCCCCAAAACAAAAGGA | | |  |
| cox3_F_EXT | 55.6 | | TGAAGCATAATTATCATCTTTTGTCT | | |  |
| cox3_R_EXT | 55.6 | | ACGATCACCAATAAACAAAAACA | | |  |
| nd3‒5_F_EXT | 55.3 | | TGATGATGGGTTTTGTTTGTTT | | |  |
| nd3‒5_R_EXT | 55.9 | | TGGAAACAAATTCTTAAATACGCA | | |  |
| nd3‒5_F2int |  | | TGGAAAGTTGGTTTGGGTTG | | |  |
| nd3‒5_F3int |  | | TGGTGGTTTAAGGAGGGTTG | | |  |
| nd3‒5_F4int |  | | AGTGATCTGACTGTATAGTGGAC | | |  |
| nd3‒5_R1int |  | | AACTAGACACACCTAATAAATCTCA | | |  |
| nd3‒5_R2int |  | | ACAGCATGACTCAACATATGA | | |  |
| nd3‒5_R3int |  | | CAACACACAAAACCAAACCCA | | |  |
| cox1_F_EXT | 63.0 | | TCTGGTATAGTTGGTGCTGGT | | |  |
| cox1_R_EXT | 58.0 | | AATCATAAATAAAAGAACGACCAGA | | |  |
| cox1_F3int |  | | TTTCTTTTGATTTTGACTTTACCTGT | | |  |
| cox1_F4int |  | | AGTTTGGACGTTGTTTTACATGA | | |  |
| cox1_R1int |  | | TGCAAATACACCAAAGTTCCAAC | | |  |
| cox1_R2int |  | | AGAACTTACACCAGAACAATGT | | |  |
| cox1_R3int |  | | CAGCAATAACCATAGTAGCAGCA | | |  |
|  | | | | | |  |
| Cycle | | Time (min:sec) | | Temperature (°C) |  |  |
| Activation | | 0:30 | | 98 |  |  |
| Denaturation | | 0:10 | | 98 | x35 |  |
| Annealing | | 0:30 | | 60** |  |  |
| Extension | | 1:00 | | 72 |  |  |
| Final extension | | 2:00 | | 72 |  |  |
| ***Start at 60°C then decrease 0.5°C every cycle until 58°C. Complete remainder of the cycles at 58°C.* | | | | | |  |
