# Supplementary material for: Population genetic analysis of Chadian Guinea worms reveals that human and non-human hosts share common parasite populations
Source: PLoS Negl Trop Dis. 2018 Oct 4;12(10):e0006747. doi: 10.1371/journal.pntd.0006747 (PMC6191157; doi:10.1371/journal.pntd.0006747)
Supplement: S2 Table — All primers specific to D. medinensis (those whose names are prefaced with “Gw”) include a 5ʹ tail that is complementary to one of 4 fluorescently tagged universal primers, following Blacket et al. (2012). Microsatellite repeat motifs are based on the draft D. medinensis genome v2.0.4. Bolded text at the 5ʹ end of the reverse primers indicates additional PIG-tailing nucleotides appended following Brownstein et al. (1996). (DOCX) [file pntd.0006747.s002.docx]

| **Suppl. Table 2.** **Microsatellite Primers and Fluorescently Tagged Universal Primer Tails.** All primers specific to *D. medinensis* (those whose names are prefaced with “Gw”) include a 5ʹ tail that is complementary to one of 4 fluorsescently tagged universal primers, following Blacket et al. [1]. Microsatellite repeat motifs are based on the draft *D. medinensis* genome v2.0.4. Bolded text at the 5ʹ end of the reverse primers indicates additional PIG-tailing nucleotides appended following Brownstein et al. [2]. | | | | |
| --- | --- | --- | --- | --- |
| Primer Names | Repeat Motif | Forward Primer (5ʹ→3ʹ) | Reverse Primer (5ʹ→3ʹ) | Observed Size Range (bp) |
| GwTRI-3C | (TAA)_15_ | CTail-AGACGTTAGCTGATTCTAGCCA | **GT**TTGATGCAACTCTGGAACCACT | 119‒183 |
| GwTET-4D | (AATA)_7_ | DTail-TCCCCTGCTTGGTTGGTTATC | **GTT**TGACTCATCGACAAGTTCACGA | 135‒255 |
| GwTRI-8D | (TAA)_21_ | DTail-CGGAGGCAGCGATCACTAAT | **GTTT**ACGTGCTTCCTGTGATCAGT | 158‒251 |
| GwTRI-9A | (ATT)_19_ | ATail-GCAGCTCTCATTTCGTGGAAAG | **GTT**TAATTCCCCACGGTCCAAATGT | 151‒213 |
| GwTRI-11D | (CTC)_12_ | DTail-GGTTCTTTCCCCCAAGAAACC | **GTTT**ACAATTTTACTCGAAAAACGTCGGA | 194‒255 |
| GwTRI-12A | (ATT)_20_ | ATail-GATGAGTGCAGCTAAACGCG | **GTT**TCGGATGAAATTCCTCGTGCA | 189‒384 |
| GwTRI-13B | (ATT)_21_ | BTail-GAGGTCTCTGTTCACTCCGC | TTTTTGCCAGTACGGGTCGT | 201‒266 |
| GwTET-14C | (TATT)_11_ | CTail-ACCGCAAAGATAATCGATAGTTTT | **GTTT**CGTTTGTCGCCCTGCTTAGT | 220‒253 |
| GwTRI-15D | (TAT)_13_ | DTail-ACGATAACAGACACAGAACGGT | **GTTT**GCGAGTCAGTTGTGCATAAGAA | 230‒295 |
| GwTET-19C | (ATTC)_10_ | CTail-ACCGCCCAATGGTATTGCAT | **GTTT**CGAAGAGGCACATTGTAATGCA | 268‒352 |
| GwTRI-20C | (TAA)_42_ | CTail-AAACATTCGTTCCTGCCACA | **GTT**TGAACAATCGCAACAATCTGACA | 185‒315 |
| GwTRI-21A | (ATT)_20_ | ATail-AGCAGTTCTAATCCATTACTTCTCG | **GTTT**AACGGCGTGTTACTGTTCCA | 259‒328 |
| GwTRI-22B | (ATT)_14_ | BTail-CAGAACGCCAAAAACTATCGGT | **GTT**TGAGCCATCATTCTGAACCTCC | 295‒417 |
| GwTRI-25B | (ATA)_22_ | BTail-TCCATCATTGTAGCGAGCCA | **GT**TTGCCGTTCCAGCAAAAAGT | 252‒431 |
| GwTRI-26C | (TTA)_21_ | CTail-ATTTCTCATGCTCCCAGGCC | TTTGCTATAAATGGCTCGCAAA | 326‒395 |
| GwTRI-27D | (AAT)_42_ | DTail-ACTCATACTGCTTAGAACGACTCT | **GTT**TGAACCATTCTGCTCAACTGC | 231‒351 |
| GwTRI-31D | (AAT)_22_ | DTail-AGTATTTCAAGGGTTTTTACAAGTCT | **GTTT**CTTCCACTTCCTGCTCGGTT | 323‒390 |
| GwTRI-32A | (TTA)_15_ | ATail-CTCTTTTGCCAAGAATTTGCCC | **GTTT**GCAGCTGCAGTTTATTTTAAATTTGT | 337‒373 |
| GwTRI-33B | (ATA)_21_ | BTail-GCTGTACTGCATCGTTTGTCA | **GTT**TCCCTTTCCTTCTTTTATTTTGCT | 333‒427 |
| GwTET-34C | (ATGG)_16_ | CTail-AGGTTGCTTGCTTTATTGGATGG | **GTTT**CCATCACCACCAGTACCTCC | 349‒436 |
| GwTET-36A | (ATTC)_13_ | ATail-GGTGCTCTTTGGAGAAAAGCG | **GTTT**GCAGTTATTAGCGATGGTGCC | 329‒422 |
| GwTRI-37C | (TAT)_22_ | CTail-AGCGCCGATGCTAAAGTTCT | **GTTT**ACGTATTTCGAGCAGTTAATGGT | 359‒450 |
| GwTRI-38B | (TAT)_19_ | BTail-TAAGCAAAGGCCGGGTTCTT | **GTT**TCATTCCAAAGAGCTTTTCTCGT | 391‒432 |
| Universal A | n/a | FAM-GCCTCCCTCGCGCCA | n/a |  |
| Universal B | n/a | VIC-GCCTTGCCAGCCCGC | n/a |  |
| Universal C | n/a | NED-CAGGACCAGGCTACCGTG | n/a |  |
| Universal D | n/a | PET-CGGAGAGCCGAGAGGTG |  |  |

**References**

1. Blacket MJ, Robin C, Good RT, Lee SF, Miller AD. Universal primers for fluorescent labelling of PCR fragments—an efficient and cost-effective approach to genotyping by fluorescence. Molecular ecology resources. 2012;12(3):456-63. doi: 10.1111/j.1755-0998.2011.03104.x.

2. Brownstein MJ, Carpten JD, Smith JR. Modulation of non-templated nucleotide addition by Taq DNA polymerase: primer modifications that facilitate genotyping. BioTechniques. 1996;20(6):1004-10. Epub 1996/06/01. PubMed PMID: 8780871.
