# Supplementary material for: Population genetic analysis of Chadian Guinea worms reveals that human and non-human hosts share common parasite populations
Source: PLoS Negl Trop Dis. 2018 Oct 4;12(10):e0006747. doi: 10.1371/journal.pntd.0006747 (PMC6191157; doi:10.1371/journal.pntd.0006747)
Supplement: S1 File — (DOCX) [file pntd.0006747.s003.docx]

# Supplemental File 1

**Derivation of Maternal Genotypes After Amplification of Pooled DNA Extractions**

*Correction for preferential amplification*

Our derivation of a single individual’s genotype from samples with a mix of maternal and larval DNA relies on comparison of the relative abundance of alleles in the mix. Prior to estimating relative allele abundance, a profile of preferential amplification was first estimated for each locus to generate a correction factor. To avoid estimating preferential amplification between alleles with different starting concentrations (e.g., maternal vs. larval), we first identified samples that likely consisted of only adult female DNA. These were samples where we did not observe parasite larvae in maternal tissue, no locus had more than 2 alleles, and the heterozygote balance at heterozygous loci suggested comparable starting concentrations of template. Heterozygote balance was calculated as the ratio of the high molecular weight (HMW) allele’s peak area to the lower molecular weight (LMW) allele’s peak area — alleles at a locus were considered balanced if the ratio was 0.6‒1.66 [1]. If an individual was borderline unbalanced at a locus or had highly disparate allele sizes and so was more difficult to evaluate, the average balance of all other loci for the individual became the deciding factor as to inclusion. Finally, using this subset of likely adult female-only heterozygotes (n = 32 individuals), linear regression of the relationship between the HMW:LMW peak area ratios and difference in allele size (as difference in repeat number) was used to generate a locus-specific correction factor. This correction factor was then applied to allele peak areas for the remainder of individuals with apparent mixed-source DNA.

*Genotype derivation*

To derive individual maternal genotypes we made the simplifying assumptions that only two individuals contributed to the mix of alleles (maternal and paternal) and that the maternal template would be present in greater starting concentrations than that of any larvae. With these assumptions in place, we first estimated the proportion of maternal DNA (*M̂x*) comprising a sample’s allelic mix for loci with four alleles, following the four-allele model in Gill et al. [2]. Relative proportions are easiest to estimate when both contributors are heterozygous because 1) relative input of each individual is typically more immediately and subjectively apparent and 2) this multi-allelic model has the least number of possible combinations to evaluate.

Next, assuming that *M̂x* is more or less equivalent across all loci for a given sample, all other loci with two or three alleles from that individual sample were evaluated following the two- and three-allele models in Gill et al. [2]. Goodness-of-fit of observed allelic proportions relative to the model’s expectation, given the estimated *M̂x,* was tested with the *Χ^2^* statistic. For samples with multiple four-allele loci, average *M̂x* was calculated and used in the model to evaluate two- and three-allele loci. In the event that a sample had no locus with four alleles, the maternal genotype was called using goodness-of-fit of observed allelic proportions to the expectations of the appropriate model (two- or three-allele) and so as to minimize the variance in estimated *M̂x* across loci.

**References**

1. Bill M, Gill P, Curran J, Clayton T, Pinchin R, Healy M, et al. PENDULUM—a guideline-based approach to the interpretation of STR mixtures. Forensic Science International. 2005;148(2–3):181-9. doi: http://dx.doi.org/10.1016/j.forsciint.2004.06.037.

2. Gill P, Sparkes R, Pinchin R, Clayton T, Whitaker J, Buckleton J. Interpreting simple STR mixtures using allele peak areas. Forensic Science International. 1998;91(1):41-53. doi: http://dx.doi.org/10.1016/S0379-0738(97)00174-6.
